# Media component bovine serum albumin facilitates the formation of mycobacterial biofilms in response to reductive stress

**DOI:** 10.1186/s12866-023-02853-6

**Published:** 2023-04-20

**Authors:** Parminder Singh Mavi, Shweta Singh, Ashwani Kumar

**Affiliations:** 1 Institute of Microbial Technology, Council of Scientific and Industrial Research, Room No 508, Sector 39 A, Chandigarh, India 160036; 2 Academy of Scientific and Innovative Research (AcSIR), Ghaziabad, Uttar Pradesh India 201002

**Keywords:** Reductive stress, Thiol-reductive stress, Drug tolerance, Mycobacterial biofilms, *Mycobacterium*, Bovine serum albumin, Cellulose sensor, IMT-CBD-mC

## Abstract

**Background:**

*Mycobacterium tuberculosis* (Mtb) forms physiologically relevant biofilms harboring drug-tolerant bacteria. This observation has brought the study of mycobacterial biofilms to the forefront of tuberculosis research. We established earlier that dithiothreitol (DTT) mediated reductive stress induces cellulose-rich biofilm formation in Mtb cultures. The molecular events associated with the DTT-induced biofilm formation are not known. Furthermore, there are only limited tools for monitoring the presence of cellulose in biofilms.

**Results:**

To decipher the molecular events associated with DTT-induced biofilm formation, we used Mtb and non-pathogenic, fast-growing *Mycobacterium smegmatis* (Msm). We observed that DTT induces biofilm formation in Msm cultures. We explored whether media components facilitate biofilm formation in mycobacteria upon exposure to DTT. We observed that media component bovine serum albumin promotes mycobacterial biofilm formation in response to DTT. Furthermore, we analyzed the composition of extracellular polymeric substances of Msm biofilms. We found that, like Mtb biofilms, Msm biofilms are also rich in polysaccharides and proteins. We also developed a novel protein-based molecular probe for imaging cellulose by utilizing the cellulose-binding domain of cellulase CenA from *Cellulomonas fimi* and fusing it to fluorescent reporter mCherry. Characterization of this new probe revealed that it has a high affinity for cellulose and could be used for visualizing cellulose biosynthesis during the development of *Agrobacterium* biofilms. Furthermore, we have demonstrated that biological macromolecule cellulose is present in the extracellular polymeric substances of Msm biofilms using this novel probe.

**Conclusions:**

This study indicates that DTT-mediated reduction of media component BSA leads to the formation of nucleating foci. These nucleating foci are critical for subsequent attachment of bacterial cells and induction of EPS production. Furthermore, this new tool, IMT-CBD-mC, could be used for monitoring cellulose incorporation in plant cells, understanding cellulose biosynthesis dynamics during biofilm formation, etc.

**Supplementary Information:**

The online version contains supplementary material available at 10.1186/s12866-023-02853-6.

## Background

Biofilm formation imparts several benefits to its residents, such as protection from various environmental stresses and anti-bacterial agents. Most prokaryotes form biofilms in their natural niches. Biofilms are primarily defined by the self-produced extracellular polymeric substances (EPS). Earlier, the role of free mycolic acids in pellicle biofilms of *Mycobacterium tuberculosis* (Mtb) was described [1]. Importantly, Mtb biofilms harbor drug-tolerant bacilli. However, environmental factors and signals that induce biofilm formation in Mtb are not defined. Another model of surface-attached biofilms was proposed by Ackart et al. It was shown that macromolecules derived from leukocyte lysate help mycobacteria form a biofilm [2]. Recently, we have demonstrated that dithiothreitol (DTT) mediated thiol reductive stress (TRS) induces biofilm formation in Mtb [3]. However, the molecular events that dictate biofilm formation in response to DTT have remained poorly defined. The high reduction potential of DTT (-0.33 V at pH 7.2) can initiate the reduction of disulfide bonds of proteins at millimolar concentrations [4]. Bovine serum albumin (BSA), one of the model proteins used to study structural changes associated with the reduction of proteins [5], is a component of supplement used for culturing Mycobacteria. BSA is a 66 kiloDalton (kDa) protein, having 17 disulfide bonds and an unpaired cysteine, making it particularly prone to reductive effects of environmental effectors. The reduction of human plasma with high concentrations of DTT (> 25 mM) can lead to the precipitation of serum albumin at ambient temperatures within 30 min [6]. Reduction of the disulfide bonds of BSA by DTT is usually followed by BSA aggregation [7], making amorphous aggregates from random disulfide bond formation upon oxidation. Whether such a mechanism plays any role in Mycobacterium DTT-induced biofilms is unknown. *Mycobacterium smegmatis* (Msm) is widely used as a model organism to understand mycobacterial physiology. Like Mtb, Msm makes pellicle biofilms [8]. However, if DTT induces biofilm formation in Msm is not known.

The nature of EPS of mycobacterial biofilms has remained an enigma. On the one hand, short-chain free mycolic acids have been proposed to constitute a significant component of mycobacterial biofilms [9, 10]. On the other hand, polysaccharides have been proposed to be a substantial component of EPS of mycobacterial biofilms [2, 3, 11, 12]. We have recently demonstrated that cellulose is a critical structural component of Mtb biofilms [3]. Cellulose is β (1 → 4) linked glucose polymer and one of the most abundant polysaccharides in nature. It serves various biological functions for bacterial cells, including structural rigidity [13], surface adhesion, conferring pathogenicity [14], etc. The central role of cellulose as a structural component of mycobacterial biofilms is supported by recent observations that overexpression of cellulase MSMEG_6752 in Msm prevents the formation of spontaneous pellicle biofilms [15]. However, whether DTT-induced mycobacterial biofilms contain cellulose is not known. Detection of cellulose in biofilms is technically challenging, and currently, calcofluor white [16] and CBM3a [17] are utilized to detect cellulose in biofilms. Calcofluor white is known to inhibit the formation of microfibrillar cellulose, while visualization of CBM3a needs probing with antibodies [18, 19], rendering it unsuitable for a variety of biological samples and live imaging. Thus, neither can be used for live imaging during biofilm formation. Accordingly, there is a need to develop tools for detecting cellulose in biofilms.

In this manuscript, we have probed whether Msm forms biofilms in response to DTT-mediated reductive stress. We have analyzed the role of media components in biofilm formation in mycobacteria. Finally, we have described the development and characterization of a new tool for detecting cellulose in bacterial biofilms and used it to explore the presence of cellulose in Msm and *Agrobacterium* biofilms.

## Results

### Thiol-reductive stress induces biofilm formation in *M. smegmatis*

**Fig. 1:**
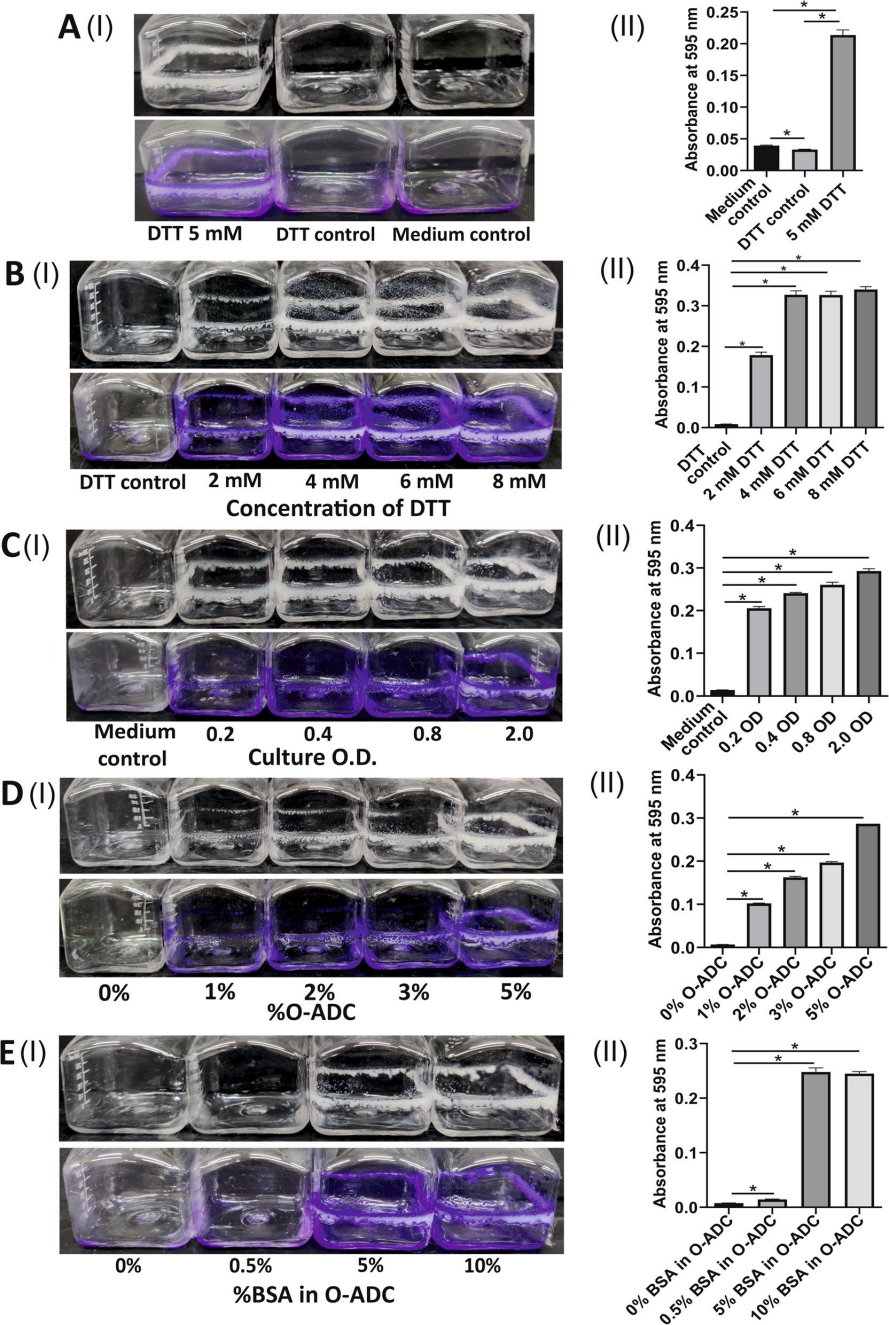
Formation of biofilm by Msm upon exposure to DTT: (**A**) (I) Culture of O.D._600_–1.0 was exposed to 5 mM DTT (left panel). In control, no DTT was added to the culture (middle panel). To media alone, 5 mM DTT was added (right panel), (II) CV assays were performed on the samples described above. (**B**) (I) Msm cultures at an OD_600_ -1.0 were exposed to a 2 mM-8 mM range of DTT, and biofilm formation was recorded after 29 h of DTT exposure observed visibly, (II) quantified with CV assay. 4–6 mM of DTT leads to the formation of biofilm. (**C**) (I) Cultures of different O.D. (0.2 – 2) were exposed to 6 mM DTT, and biofilm formation was recorded after 29 h of DTT exposure observed visibly, (II) quantified with CV assay. Biofilm formation was observed at all O.D.s. (**D**) (I) Msm culture of OD_600_—1.0 was raised in a medium containing different concentrations of O-ADC (as indicated below the inkwell bottle). These cultures were exposed to DTT for 29 h and observed visibly, (II) quantified with CV assay. (**E**) (I) 7H9 medium was supplemented with reconstituted O-ADC having a variable concentration of BSA (concentration of BSA is denoted as % below the inkwell bottles), keeping the rest of the components constant. Bacterial culture was raised in these media and exposed to 6 mM DTT. Biofilm formation was facilitated in a BSA-dependent manner and was observed visibly, (II) quantified with CV assay. The column bar graphs were plotted using GraphPad Prism 8. Data represent mean ± SEM from 3 independent experiments. Statistical significance was determined using Student’s *t*-test, * indicates a *p*-value of < 0.05

We have recently demonstrated that Mtb and other mycobacterial species form a biofilm in response to TRS [3, 20]. Since Msm is the model organism for studying mycobacterial physiology, we analyzed whether TRS also induces biofilm formation in Msm. We have used DTT to induce TRS in the Msm cell. To test this, we exposed logarithmic cultures of Msm in 7H9 medium supplemented with O-ADC (containing oleic acid-25 µg/mL, dextrose-1 mg/mL, catalase-2 µg/mL and BSA-2.5 mg/mL), to 5 mM DTT. Similar to Mtb, exposure of Msm cultures to DTT induced biofilm formation within 29 h of exposure (Fig. 1A (I)). The quantitation of the visibly observed phenomenon was performed using crystal violet (CV) assay, and statistical differences were observed between 5 mM DTT, DTT control, and medium-only control without cells (medium control) (Fig. 1A (II)). To analyze the exact concentration of DTT required for biofilm formation, Msm cells were exposed to a concentration gradient of DTT (ranging from 2 to 8 mM). We observed that 4–8 mM DTT induces biofilm formation in Msm (Fig. 1B (I)). The quantitation of the visibly observed phenomenon was performed using CV assay and statistical differences were observed between control, 2, 4, 6, and 8 mM DTT (Fig. 1B (II)). Unlike the pellicle biofilms, these biofilms stringently adhered to the inkwell bottles' walls at the liquid–air interface. Similar to Mtb, (Supplementary 2) exposing standing cultures of Msm to TRS-induced mat-like submerged biofilms strongly adherent to the substratum at the bottom of the culture vessel (Supplementary 3). Exposure to a gradient of DTT concentration suggests that 4 mM of DTT is sufficient to induce biofilm formation in the standing cultures of Msm. Furthermore, the biofilms are luxuriant beyond 6 mM DTT concentration. Thus, we have used 6 mM DTT for the rest of the experiments. We also tested if biofilm formation depends on the initial O.D. of the culture and treated Msm cultures to 6 mM DTT at an O.D._600_ of 0.2, 0.4, 0.8, and 2.0 and recorded biofilm formation after 24 to 29 h. Medium only without cells (medium control) was taken as a control in the experiment. We observed biofilm formation in cultures of varying O.D. in response to DTT whereas, absent in the medium control (Fig. 1C (I)). However, it must be noted that thicker biofilms were formed in cultures of higher O.D._600_. The quantitation of the visually observed phenomenon was performed using CV assay, and the statistical difference was observed between control, 0.2, 0.4, 0.8, and 2.0 OD (Fig. 1C (II)). Biofilms were also formed in 24 well plates to quantify the above-mentioned phenomenon to observe a more quantifiable statistical difference compared to inkwell bottles (Supplementary Fig. 1 A-C (I, II)).

### BSA is critical for TRS induced mycobacterial biofilm formation

Dubos and Middlebrook formulated broth-based media for culturing mycobacteria in 1947 [21]. They supplemented the broth medium with oleic acid, BSA, dextrose, and catalase for enhanced growth. BSA was added as a protective agent due to its property of binding to toxic free fatty acids, while dextrose acts as an energy source and catalase neutralizes oxidative stress. Ojha and Hatfull have earlier demonstrated that micronutrient iron plays a critical role in forming Msm pellicle biofilm [22]. However, the role of media supplement O-ADC in biofilm formation has not been examined so far. Although Msm can grow without O-ADC supplement, we cultured Msm in a medium containing O-ADC, for comparative analysis with Mtb, which requires OADC supplementation. We subjected Msm cultured in media containing different O-ADC (1%, 2%, 3%, and 5%) concentrations to 6 mM DTT. Interestingly, we observed a decrease in biofilm formation in cultures with the reduced O-ADC supplement (Fig. 1D (I)). The quantitation of the phenomenon was performed using CV assay, and statistical differences were observed between control, 3%, and 5% O-ADC (Fig. 1D (II)). Since BSA has 17 disulfide bonds that can be reduced by DTT [23] and DTT treatment leads to BSA aggregation [5, 7], we examined the possibility that BSA may be aiding the formation of biofilms by mycobacteria under reductive stress. Usually, the O-ADC contains 5% BSA (weight/volume). The O-ADC supplement was reconstituted to have variable concentrations of BSA (0%, 0.5%, 5%, and 10%) but constant concentrations of the rest of the components. Msm cultures were raised in a 7H9 medium supplemented with the reconstituted O-ADC. Interestingly, we observed that the amount of biofilm formed correlated well with BSA concentrations in the O-ADC supplement (Fig. 1E (I)). The quantitation of the phenomenon was performed using CV assay and the statistical difference was observed between control, 5% and 10% BSA in O-ADC (Fig. 1E (II)). This was true for the biofilms at the air–liquid interface in shaking cultures and the surface adherent biofilms in standing cultures. Mtb biofilms (Supplementary Fig. 2 (I) and (II)) and Msm biofilms (Supplementary Fig. 3(I) and (II)) developed under similar conditions showed a similar response. The adherent material amount was visualized and quantitated by CV staining (Supplementary Fig. 2 (II) and 3 (II), respectively) [24]. However, in the absence of bacterial cells, the BSA did not form visible aggregates/films at 24 h, indicating a specific role of the mycobacterial cells in the process (Fig. 1A). Biofilms were also formed in 24 well plates to quantify the above-mentioned phenomenon to observe a more significant statistical difference compared to inkwell bottles (Supplementary Fig. 1 D-E (I, II)).To ascertain whether the presence of secreted proteins from Msm has a significant role in the aggregation of BSA on exposure to DTT, we subjected medium alone (7H9-5% O-ADC, with and without Tween-80 additive, which could influence the aggregation of BSA) and conditioned medium (filtered supernatant medium with O-ADC, cultured with Msm) to 6 mM of DTT and recorded the change in intrinsic fluorescence of BSA as a function of time over 24 h. This intrinsic fluorescence (excitation at 280 nm) arises from the tryptophan residues. BSA’s intrinsic fluorescence has been shown to decrease with time on conformational change, hydrophobic exposure, and aggregation on exposure to DTT [7]. We observed that intrinsic tryptophan fluorescence shows a steady decrease with time (Supplementary Fig. 4), possibly due to the denaturation of proteins, as has been demonstrated for BSA. However, a comparable total downshift in autofluorescence was observed for the conditioned medium as well, indicating that the medium utilized by Msm for growth containing a mix of proteins secreted by the bacteria and BSA collectively shows a similar reduction by DTT, as seen by the change in intrinsic tryptophan fluorescence changes. The presence of Tween 80 as an anti-clumping agent does not offset this effect (Supplementary Fig. 4).

### Polysaccharides are a component of the Msm biofilms

**Fig. 2:**
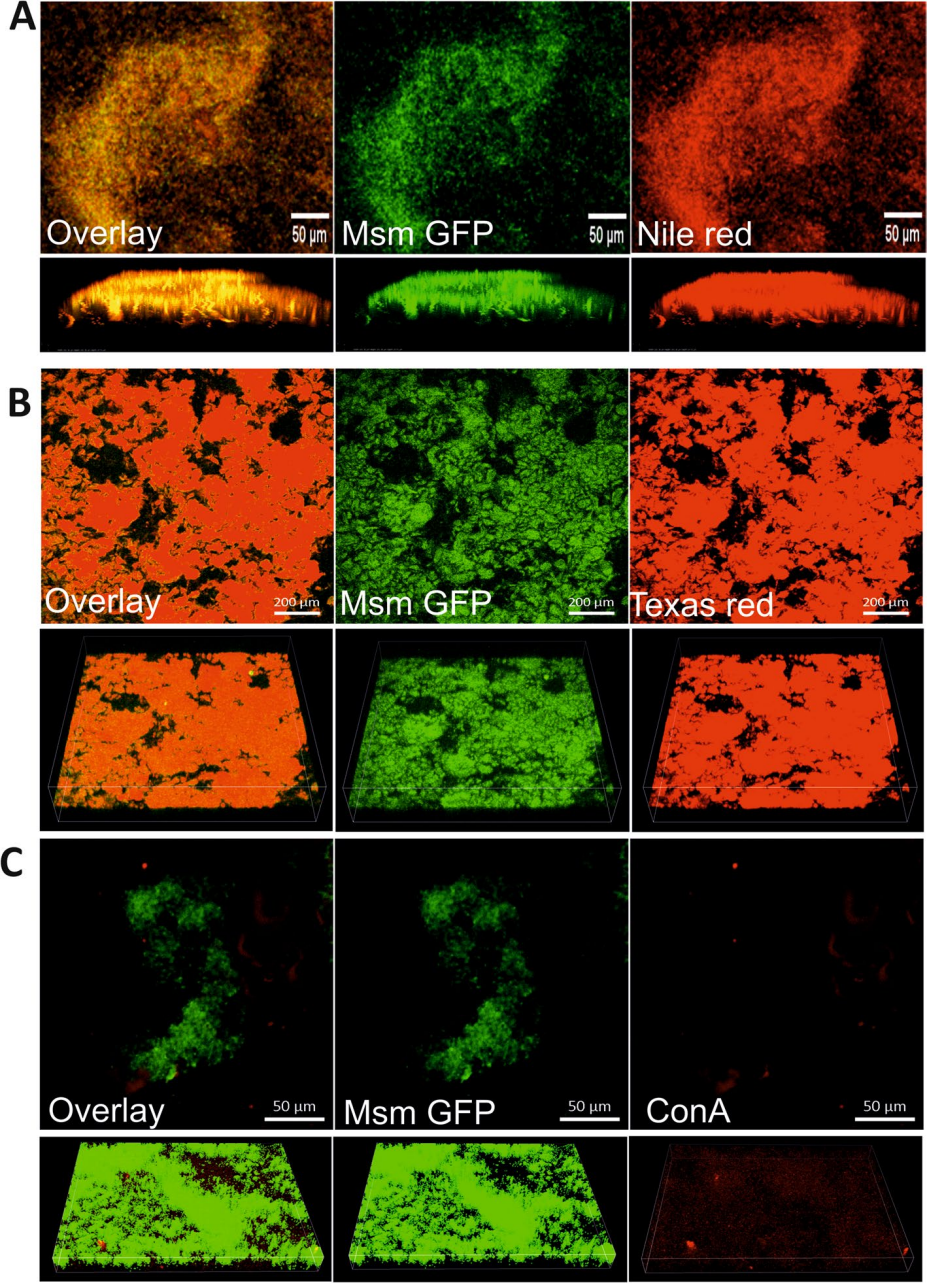
DTT-induced Msm biofilms are rich in polysaccharides. Msm overexpressing GFP were exposed to 6 mM DTT for 29 h to induce biofilm formation. Biofilms were then stained with specific fluorophores, namely, (**A**) Nile red (1 mM) for staining the lipids, (**B**) Texas red Hydrazide (0.5 mg/ml) for staining polysaccharides, (**C**) lectin ConA (200 μg/ml) for staining the α-mannopyranosyl and α-glucopyranosyl-rich polysaccharides. Stained biofilms were then analyzed using CLSM (Scale bar: A and C-50 µm, B-200 µm). Lower panels in **A**-**C** show the 3-D image of the biofilms. Images are representative of three independent experiments

Earlier studies have demonstrated that polysaccharides and lipids constitute the significant components of Mtb biofilms. However, the composition of EPS in the DTT-induced Msm biofilms is unknown. Thus, to test the composition of Msm biofilms, we transformed Msm with episomal plasmid pMV762 [25] carrying the gene for enhanced green fluorescent protein (eGFP) and stained the TRS biofilms formed by the modified strain for the presence of lipids and polysaccharides. Particularly, Nile red for staining lipids (Fig. 2A) and Texas Red Hydrazide for polysaccharides (Fig. 2B). We observed that Texas red Hydrazide profusely stained the DTT-induced Msm biofilms, suggesting that polysaccharides constitute a significant component of these biofilms. We also analyzed whether lectin Con-A stains α-mannopyranosyl and α-glucopyranosyl-rich polysaccharides could stain these biofilms, although not as profusely as Texas Red Hydrazide (Fig. 2C). These observations indicate that biofilms formed contain extracellular polysaccharides. Earlier, we identified cellulose in Mtb biofilms [3]. Therefore Msm, a close homolog of Mtb, may also use cellulose as a component of biofilms.

### Development and characterization of a new probe for cellulose detection in biofilms

**Fig. 3:**
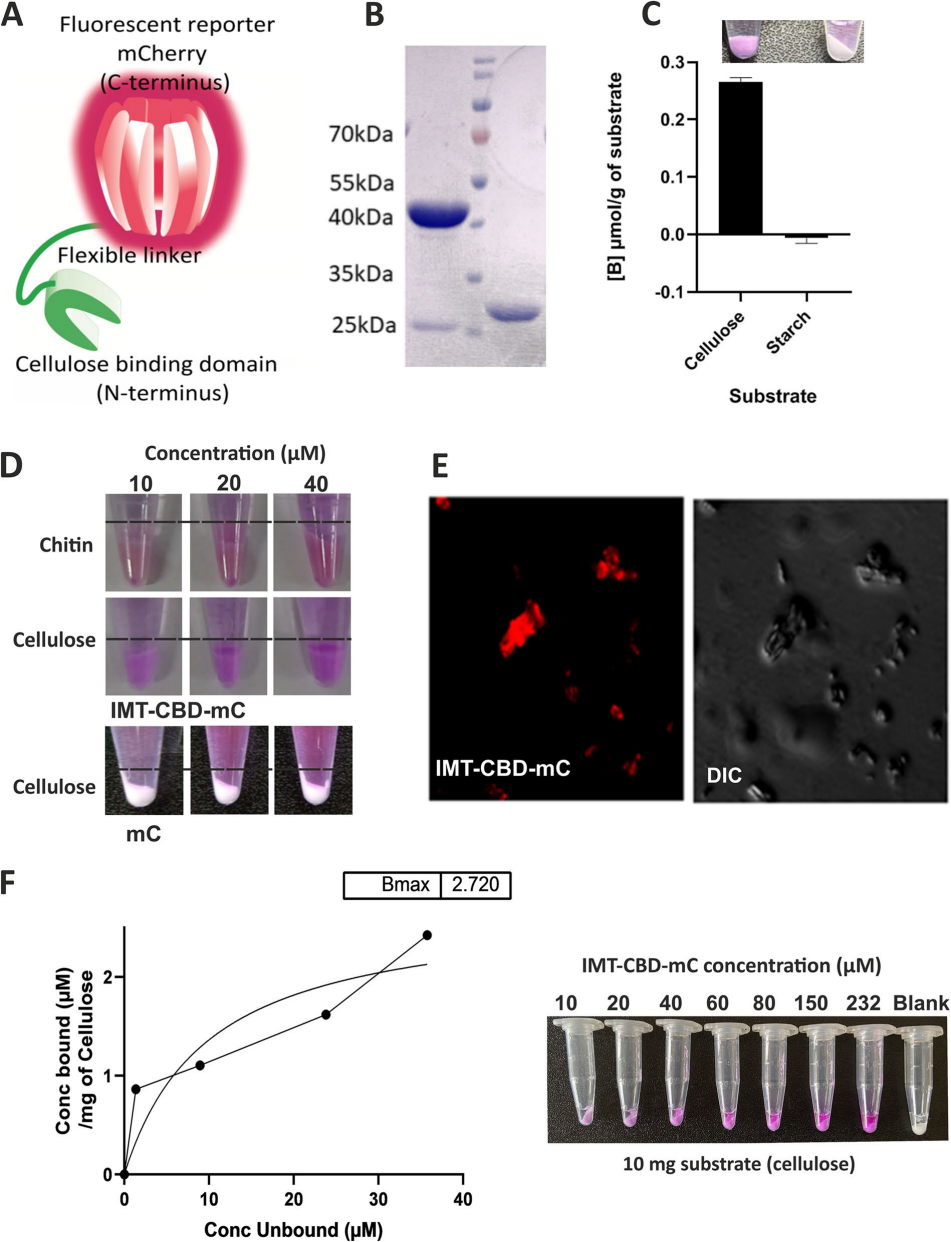
Development and characterization of a new tool for staining cellulose in biofilms. (**A**) Design of IMT-CBD-mC. In this probe, CBD of CenA from *C. fimi* is linked to mCherry through an octa-glycine linker. (**B**) IMT-CBD-mC (left to the marker) and mCherry (right to the marker) were purified after overexpression in *E. coli*. Molecular weights on the marker are shown to the left, and the middle lane represents the marker (**C**). This probe was used to stain cellulose (left tube in the panel) and starch (right tube in the panel). 10 µM of the protein was incubated with 10 mg of cellulose and starch and incubated at 4˚C overnight. The amount of protein bound to the substrate ([B] µmol/g of the substrate) was quantitated using fluorescence from mCherry. A representative image of the IMT-CBD-mC binding to either substrate is shown above the graph (**D**) Binding of IMT-CBD-mC to chitin and cellulose to increasing concentrations of the protein (left to right). To test if the binding to cellulose is due to CBD and not due to mCherry, cellulose was stained with either of the purified proteins. Only IMT-CBD-mC was observed to bind to cellulose and chitin (i). (**E**) Purified IMT-CBD-mC was used to stain cellulose and visualized using fluorescence microscopy. The left panel shows the fluorescence of IMT-CBD-mC bound to cellulose, and the right panel shows a differential interference contrast (DIC) image of the stained cellulose crystals. (**F**) Solution depletion isotherm of IMT-CBD-mC against cellulose indicates that the IMT-CBD-mC probe effectively binds cellulose as its substrate. Y-axis ([B] µmol/g of the substrate) indicates bound protein per gram of cellulose; an X-axis indicates free ([F]) protein in the solution after binding. The Bmax value of the fitted curve is indicated at the top of the graph. A graph with a closed circle represents the actual data, and a line curve shows the mathematical model fitting with the data

Earlier, Wyk et al*.* [15] have suggested that cellulose may also be a component of pellicle biofilms of Msm. Therefore, we examined if cellulose is present in an intact Msm biofilm. Currently, calcofluor white, CBM3a-peptide antibodies, and luminescent oligothiophenes [26] are employed for staining cellulose. Nonetheless, newer tools are desired for studying the real-time formation of biofilms. Towards this, we engineered a cellulose-specific probe by fusing the cellulose-binding domain (CBD) of CenA of *Cellulomonas fimi* (*C. fimi*) with the fluorescent protein mCherry (Fig. 3A). CenA of *C. fimi* contains a CBD separated by a rigid linker from the cellulase domain [27]. The N-terminally 6xHis-tagged fusion product was named IMTECH-CBD-mCherry (IMT-CBD-mC) and was purified after overexpression in *E. coli* with IMAC Ni–NTA chromatography (Fig. 3B). We also purified mCherry without CBD as a control for specificity (Fig. 3B). Next, we analyzed the specificity of IMT-CBD-mC for binding to glucose polymers such as cellulose and starch (S4180, Sigma), wherein glucose is linked through β (1 → 4) and α (1 → 4) linkages respectively. We observed that the IMT-CBD-mC probe binds cellulose and does not bind to starch (Fig. 3C). These observations indicate the probe's specificity to bind β-1, 4 linkage in polysaccharides. Both cellulose and chitin carry β-1, 4 linked units, and are known to be bound by the CBD domain [28]. To test the ability of IMT-CBD-mC to stain cellulose and chitin in general and bacterial cellulose in particular, we used the purified protein to bind to microcrystalline cellulose and chitin. We found that the IMT-CBD-mC could bind to the substrates stably (Fig. 3D). mCherry alone was also tested to check if the binding observed with IMT-CBD-mC arose due to mCherry alone (Fig. 3D). We observed that mCherry alone was unable to bind cellulose. These observations validate the specificity of IMT-CBD-mC-cellulose interaction. We next tested the probe's suitability to visualize microcrystalline cellulose after staining with IMT-CBD-mC, using fluorescence microscopy. Cellulose was effectively stained by the IMT-CBD-mC probe (Fig. 3E). To further characterize the binding affinity of IMT-CBD-mC-, we employed solution depletion isotherm analysis. A B-max of 2.720 for binding of IMT-CBD-mC with cellulose was observed through solution depletion isotherm (4˚C) (Fig. 3F).

### IMT-CBD-mC could be employed for live imaging of biofilms

**Fig. 4:**
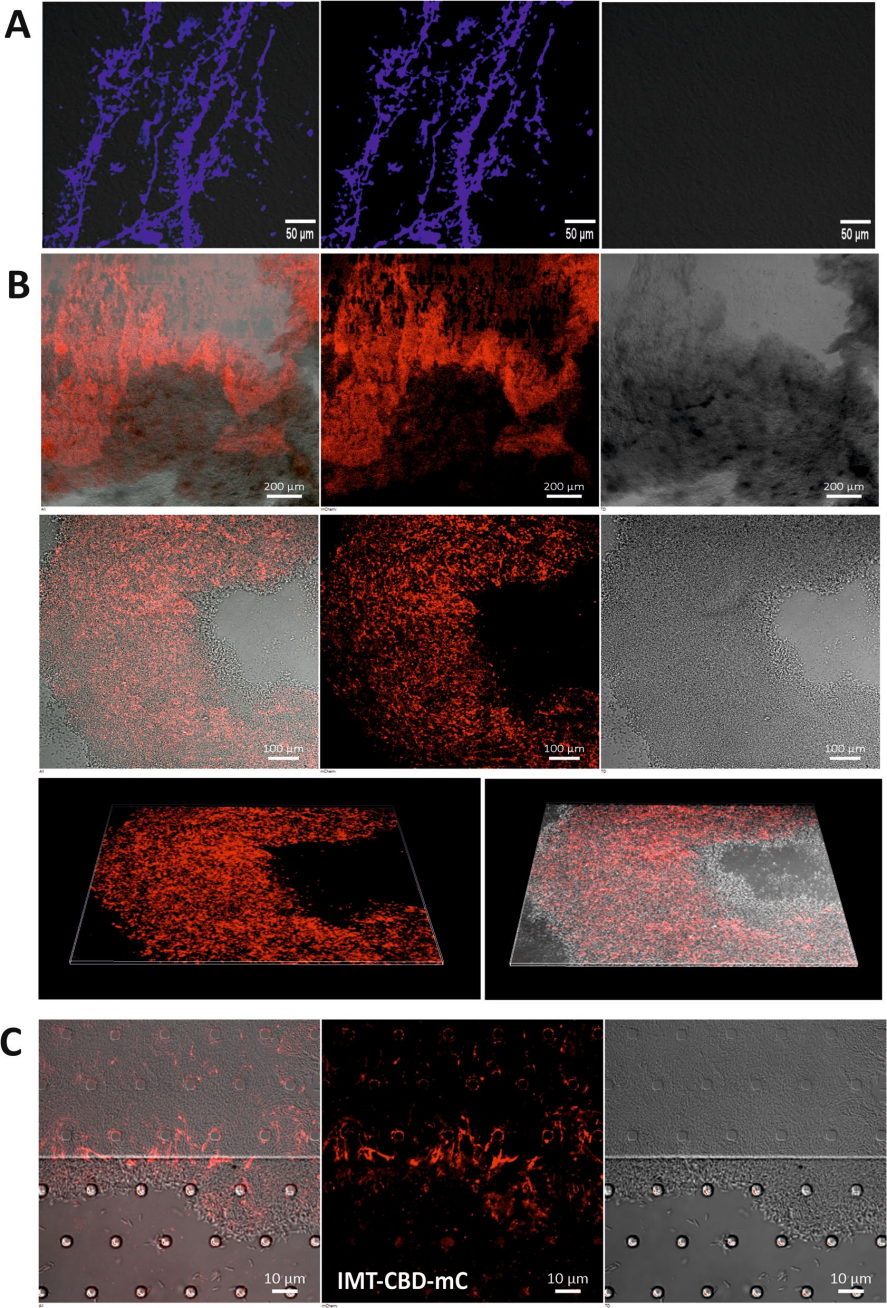
Visualisation of cellulose in *Agrobacterium tumefaciens* C58 biofilms with IMT-CBD-mC using CLSM. (**A**) Calcofluor white staining of coverslip biofilms of *A. tumefaciens* C58 raised in TSB medium. The bar indicates a length of 10 µm scale. (**B**) Purified IMT-CBD-mC was used to stain coverslip biofilms of *A. tumefaciens* C58 grown in a TSB medium. A 2-dimensional scan of the biofilm reveals staining of bacterial cellulose by IMT-CBD-mC. The top panel is at 40 × magnification (bar-200 µm), while the middle panel is at 100 × magnification (bar100 µm). 3-dimensional reconstruction for the biofilm stack (lowest panel) shows the structural morphology of the biofilm along with visualization of cellulose (red) vis-à-vis bacterial cells (DIC). (**C**) *A. tumefaciens* C58 cells in the microfluidic chamber were stained with IMT-CBD-mC and visualized using CLSM. The left panel is the merge, the middle panel IMT-CBD-mC, and the right panel is the (DIC) image. The bar indicates a length of 10 µm scale. Images are representative of three independent experiments

Next, we examined whether IMT-CBD-mC could be used for imaging biofilm formation. Towards this, *Agrobacterium tumifaciens* C58 (MTCC 609) (hereafter referred to as AT C58) was utilized. AT C58 is well known to produce cellulose-rich biofilms [29, 30]. AT C58 coverslip biofilms were raised in Tryptic Soy Broth (TSB). Calcofluor white stained coverslip biofilms of AT C58 were visualized under a fluorescence microscope to visualize the cellulose (Fig. 4A). In agreement with this, we found that the IMT-CBD-mC stained AT C58 biofilms (Fig. 4B), showing that it can reliably be used as a tool to probe cellulose in biofilms. To test this tool's usability with live-cell imaging, we added 5 µM protein to the culture medium of AT C58. We imaged the staining of cellulose produced by the bacterium in microfluidic chambers (Fig. 4C). We found that the protein stain can be successfully used to visualize cellulose production during biofilm formation. Notably, the presence of IMT-CBD-mC in the culture medium does not hinder biofilm formation and thus facilitates live imaging. Furthermore, visualization of the IMT-CBD-mC does not require fixing of samples and then staining with antibodies. Therefore, this novel tool is suitable for live imaging. In summary, IMT-CBD-mC is a specific tool that can be used in versatile ways to identify cellulose in situ in biological materials, particularly biofilms.

### Staining M. smegmatis biofilms with IMT-CBD-mC

**Fig. 5:**
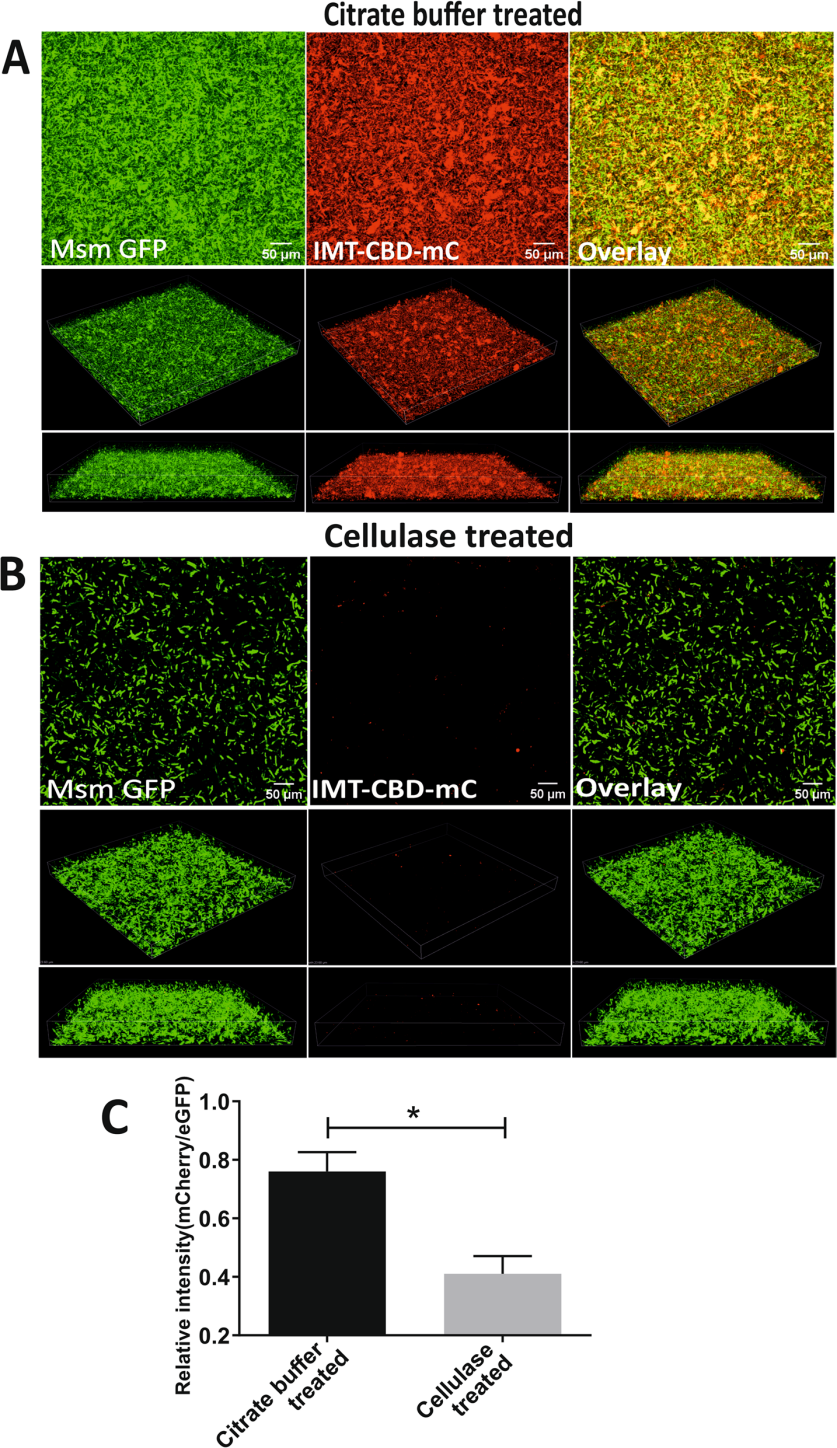
DTT-induced biofilms of Msm-eGFP are disrupted by cellulase treatment and visualized using IMT-CBD-mC in CLSM. Representative images of DTT induced biofilms of Msm overexpressing GFP (6 mM DTT conc. for 29 h) (**A**) citrate buffer treated (**B**) cellulase treated, stained with IMT-CBD-mC (Scale bar: A, B 50 µm (upper panel). The upper panels show the 2D images of the biofilms, whereas the two lower panels show the Z stack projections of the biofilm (in both A and B). (**C**) The relative intensity of IMT-CBD-mC in panels (**A**) and (**B**) from three independent experiments was quantitated using Imaris V 9.2 software. The column bar graphs were plotted using GraphPad Prism 8. The data presented in (**C**) is expressed as the mean (± s.e.m.). Statistical significance was determined using Student’s *t*-test,* indicates a *p*-value of < 0.05. (**A**, **B**) Representative of at least three independent biological experiments performed in triplicate

After engineering IMT-CBD-mC and establishing its ability to stain bacterial biofilms, we questioned if Msm biofilms possess cellulose and whether IMT-CBD-mC could detect it. Towards this, we constructed Msm strain overexpressing eGFP. Msm-eGFP cultures at an O.D._600_ of 1 were exposed to DTT to enhance the biofilm formation. Subsequently, adherent biofilms of Msm-eGFP were stained for cellulose using IMT-CBD-mC and analyzed using confocal laser scanning microscopy. Interestingly, the IMT-CBD-mC stained the biofilm matrix, indicating the presence of cellulose in the Msm biofilms (Supplementary Fig. 5). To further confirm our findings, we treated the Msm biofilms with cellulase enzyme and observed that upon treatment with cellulase, Msm biofilms were disintegrated into bacteria cell suspension (Fig. 5 A-C). Furthermore, the IMT-CBD-mC staining was diminished in the cellulase treated panel compared to one treated only with citrate buffer. These observations suggest that Msm biofilms contain substantial cellulose, and IMT-CBD-mC is specific for detecting cellulose in mycobacterial biofilms.

## Discussion

**Fig. 6:**
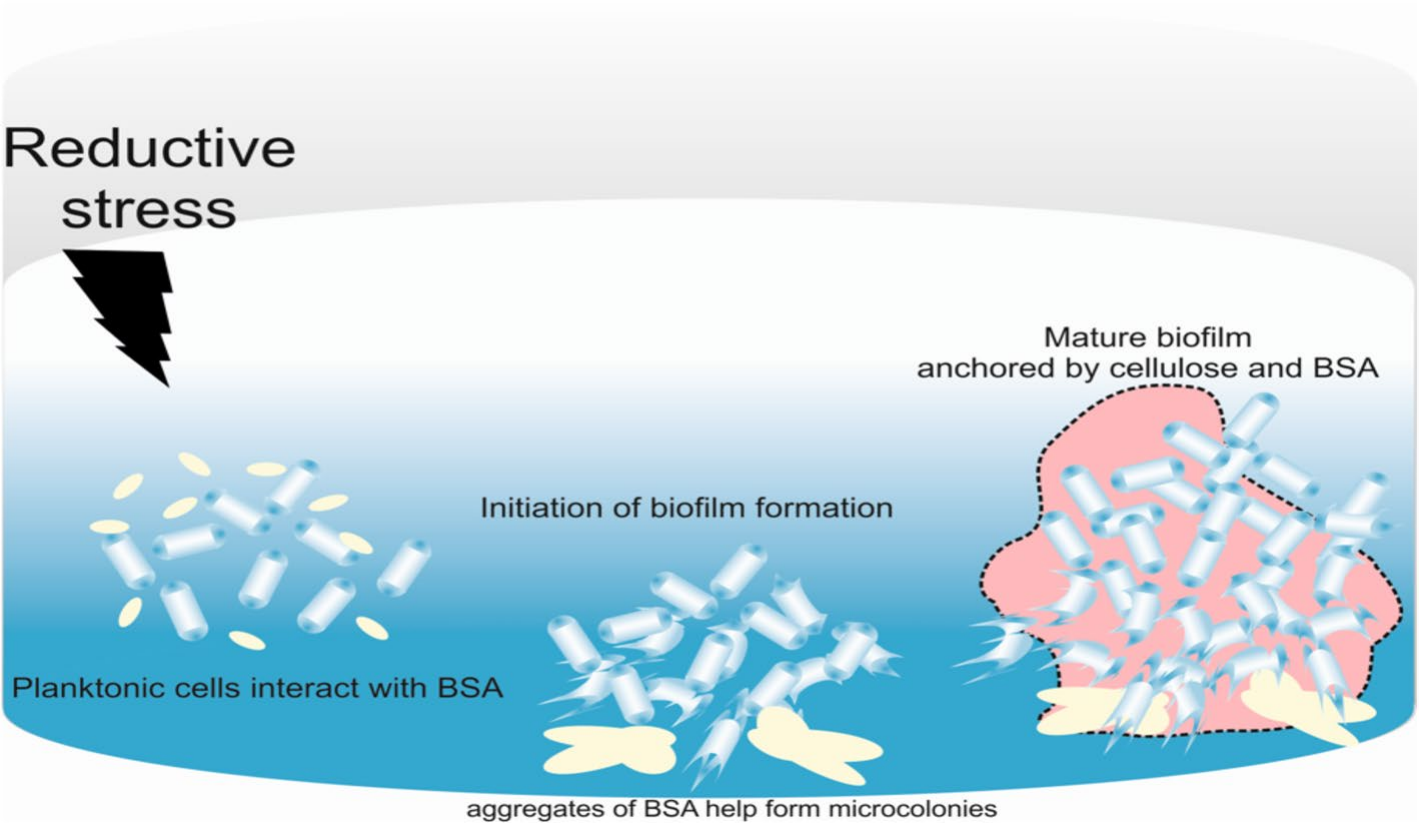
Model depicting the formation of biofilm by mycobacteria. BSA in the culture medium possesses several disulfide bonds, which are reduced by adding DTT. With the reoxidation of these reduced bonds, aggregate formation occurs, which leads to the formation of microscopic niches for the bacterial cells to initiate biofilm formation. Notably, intracellular TRS is required for biofilm formation as β-mercaptoethanol does not induce biofilm formation. Mycobacteria utilize cellulose to bind to the substratum and form a dense biofilm at maturation

DTT is used to study thiol reductive stress in bacterial cells and is known to induce biofilm formation in Mtb cultures [3]. The molecular events underlying the formation of mycobacterial biofilms in response to DTT have remained poorly understood. Here, we utilized Mtb and Msm to delineate the role of media components in biofilm formation. Data presented in this study suggest that BSA, a component of media supplement, is critical for biofilm formation in response to DTT. We believe that BSA (a component of O-ADC) could react with DTT and may form microaggregates, which could act as a nucleation site for biofilm formation in mycobacterial cultures (Fig. 6). We have also developed a new probe for detecting cellulose in biofilms. Using this tool, we have detected cellulose in the biofilms of *Agrobacterium* and *Mycobacterium*.

One of the critical findings of this manuscript is defining the role of O-ADC supplement in mycobacterial biofilm formation. Several additives are utilized to improve the growth and dispersion of mycobacterial cells. Dextrose and glycerol aid the growth of Mtb by providing readily available carbon sources, and catalase destroys toxic peroxides. Wetting agents such as detergents are often added to maintain diffused bacterial cultures instead of aggregates. Commonly, Tween 80 is used for this purpose in mycobacterial culturing. Also, oleic acid helps in supplementing the nutritional requirements of Mtb. The addition of Tween 80 and oleic acid might be counterproductive, as unesterified fatty acids from Tween 80 are lethal to Mtb at concentrations required for proper dispersion of aggregates [31]. Davis and Dubos observed that BSA provides a protective effect against this toxicity [21], and since then, it has been standard practice to use BSA as a supplement for culturing Mtb. The exact mechanism of BSA aided detoxification has not been worked out, though it was shown that BSA binds carboxyl groups of fatty acids and increases their solubility [32]. Subsequently, it has been proposed that fatty acid-free BSA (or BSA fraction V) helps in the binding of fatty acids and helps increase the wetting of the surface of bacteria, and increases access to nutrients such as oleic acid. Besides this, BSA may have a nutritional effect on Mtb, though not by supplying amino acids [21, 31]. Significantly, DTT reduces disulfide bonds in BSA and induces the formation of microaggregates [6, 7]. We believe that such microaggregates could act as nucleation sites to form mycobacterial microcolonies observed with Mtb of Msm. These microcolonies could then grow into macro-colonies, and thus BSA could facilitate biofilm formation in the mycobacterial cells (Fig. 6). However, it must be noted that the denaturation of BSA is not the sole factor for biofilm formation. Another reducing agent, β-mercaptoethanol, capable of reducing BSA thiols [33], does not induce biofilm formation in Mtb [3]. Importantly, β-mercaptoethanol is unable to enter mycobacterial cells and is not able to generate intracellular TRS. Thus, besides the reduction of BSA, another critical requirement for induction of biofilm formation in intracellular TRS. In summary, this manuscript identifies that there are two critical requirements for mycobacterial biofilm formation. Firstly, nucleation sites are required for biofilm formation. The aggregation of BSA by DTT induces the formation of microaggregates. Secondly, programming of the genetic pathways for synthesizing extracellular polysaccharides thus enables biofilm formation. This programming is induced by intracellular thiol reductive stress. Physiologically for Mtb biofilm formation in vivo, tissue damage caused by the host immune system may provide the nucleation site, and thiol reductive stress induced by reagents like cellular GSH may induce biofilm formation.

Besides establishing the role of media components in mycobacterial biofilm formation, we have also developed a new tool for examining the presence of cellulose in biological samples. However, Calcofluor white and CBM3a are currently available to detect cellulose in biological samples. Calcofluor white binds beta-glucans with high affinity, inhibiting cellulose microfibrils formation [18, 19]. Thus, it is not suitable for experiments wherein cellular physiology is output along with the detection of cellulose. Furthermore, visualization of Calcofluor white depends upon the availability of UV lasers not available with most of the fluorescent microscopes. On the other hand, while CBM3a binds cellulose and chitin with high affinity, it is detected using fluorescently labeled antibodies. Detection with antibodies requires fixing samples and often requires permeabilization of host cells. Thus, CBM3a and Calcofluor white cannot be used in experiments requiring temporal resolution. The probe described in this study relies on the CBD of *C. fimi,* having a high affinity for cellulose/chitin [27, 34, 35]. For detection, this probe utilizes mCherry fused at the C-terminal of CBD. Thus, this probe could be used in live-cell imaging experiments. Indeed, this was the case, and we were able to use this probe to visualize the formation of biofilms of *Agrobacterium*. Another important finding of the study was the demonstration that cellulose is a component of Msm biofilms like Mtb biofilms. Towards this, we have utilized IMT-CBD-mC and demonstrated that cellulose is a key component of the EPS of Msm biofilms. These findings are consistent with the observation that the overexpression of cellulase inhibits the spontaneous formation of pellicle biofilms [15].

In summary, this study has delineated the role of media components in forming mycobacterial biofilms in response to DTT-mediated thiol reductive stress. Furthermore, this study describes the engineering of a novel probe to detect cellulose in bacterial biofilms.

## Conclusions

The role of media components in the formation of mycobacterial biofilms is largely unexplored. Only one earlier study suggests that mycobacterial biofilm formation is dependent on the presence of iron in the culture medium [22]. In this manuscript, we have explored the role of media components, including the O-ADC supplement, in biofilm formation. Using several lines of evidence, we have demonstrated that BSA, a component of the O-ADC supplement used in culturing Mtb, is critical in biofilm formation in response to DTT. Furthermore, we have observed that cellulose is a vital component of the Msm biofilms similar to Mtb. We have also developed a new biological sensor for cellulose. This new probe was utilized to demonstrate the presence of cellulose in *Agrobacterium* and Msm biofilms.

## Methods

### TRS induced biofilm formation by Msm and Mtb

Biofilms were formed by treating cultures to DTT (Sigma) as previously described for Mtb [3]. Msm (mc^2^ 155) was cultured in Middlebrook 7H9 broth supplemented with 1, 2, 3, 5% O-ADC, 0.2% glycerol (G0040, Rankem) and 0.05% Tween 80 (MP, 103,170). For preparation of O-ADC we prepare O-ADC stock solution (1 L) constituting of Oleic acid (01,008, Sigma) 0.6 ml, Bovine serum albumin fraction V (GRM105, HiMedia) 50 gm, Dextrose (194,024, MP) 20 gm, sodium chloride (1.93606.0521, Merck) 8.5 gm, catalase (C1345, Sigma) 0.03 gm and is filter sterilised using 0.2 micron filter. Briefly, cultures of Mtb (H37Rv) and Msm (mc^2^ 155) at indicated O.D. in 7H9 broth with the indicated concentration of O-ADC supplement was subjected to DTT as indicated (2, 4, 6, and 8 mM) (from a 1 M filtered stock) and cultured for 24 h, 90 r.p.m. at 37 °C temperature. 50 µg/mL of Hygromycin B was added to the cultures where appropriate. 25 µg/mL kanamycin was added where appropriate. For inkwell bottle-related experiments, cultures were grown in 10 ml media with required experimental conditions and for 24-well plates, 1 ml of culture was transferred to each well, and DTT induction was given as per the experimental conditions. Biofilm formation was also studied in 96-well plates. For biofilm formation in 96-well plates, DTT was added to the 200 µl culture. Biofilms were also formed in 8-well-chambered slides for confocal microscopy. For inoculation in 8-well chambered slides, cultures of OD 0.8–1 were taken and 400 µL was transferred to each well, and DTT induction with 6 mM DTT was given for 29 h at 37 °C.

### Intrinsic fluorescence measurements

Fluorescence measurements were performed by collecting a 200 µL sample of the culture medium supernatant after filtration (0.2 microns) and read in a transparent bottom 96-well plate at room temperature in a BioTek® hybrid fluorimeter. The samples were diluted with 1X Phosphate buffered saline (PBS) to a final protein concentration of 5 µM BSA for fluorescence experiments. Intrinsic fluorescence spectra for medium proteins were collected in the 300–400 nm range with excitation at 280 nm. Data points represent an average of three biological replicates at each time point.

### Cloning CBD mCherry

*E. coli* Top10 cells were used for all cloning experiments. Codon optimized sequence of IMT-CBD-mC for expression in *E. coli* was obtained from Genscript® with the CBD of *Cellulomonas fimi cenA* (N-terminal 115 amino acids coding gene sequence) placed upstream to, and separated from *mCherry* gene sequence, by 8X glycine linker GGTGGTGGTGGTGGCGGAGGTGGT and a HindIII site, flanked by BamHI and XhoI 5’ and 3’ terminally respectively, in pUC57 (Genscript, Piscataway, N.J., USA) vector. The sequence was subcloned into a pET28a (Novagen, Merck KGaA, Darmstadt, Germany) vector and was used to clone IMT-CBD-mC in pET28a to obtain pET28a-CBD-mC.

### Protein expression and purification

*E. coli* BL21(DE3) cells harboring pET28a-CBDmC or pET28a-mcherry were inoculated in 10 mL L.B. with Kanamycin (50 µg/mL) for growth overnight at 37 °C (220 r.p.m) till saturation. 10 mL of saturated culture was then used for inoculating 1 L of L.B. (Kanamycin) medium, which was subsequently induced with 0.5 mM IPTG at O.D._600_ of 0.4–0.6 and cultured at 24 °C for 8 h.

For protein purification, cultures were harvested by centrifugation at 6010 rcf at 4 °C and lysed by sonication (Sonics ultra-cell sonicator, model no VCX750 was used at 20% amplitude/5 s on/15 s off/1 h total) in protein purification buffer (PPB: Tris–HCl 50 mM pH 7.5, 0.3 M NaCl, 10% Glycerol) with imidazole (20 mM).

The lysate was cleared by centrifugation at 24,000 X g for 20 min at 4 °C, and the resulting supernatant was used for IMAC-based purification with Ni–NTA resin (P6611, Sigma) equilibrated with PPB with 20 mM Imidazole. Bound protein was washed with 10 column volumes (29,922–5 ml, Thermo scientific) of PPB with 35 mM imidazole and eluted with PPB with 250 mM imidazole. Purified protein was found to be stable when stored at 4 °C.

### Substrate binding assays

Binding of CBD-mC or mCherry to microcrystalline cellulose (CC) (435236, Sigma) and chitin (C9752**,** Sigma) was performed in Tris–Cl 50 mM (pH 7.5), 0.3 mM NaCl. 10 mg of CC or chitin was mixed end-over-end with increasing amounts of the protein in 500 µL or 1 mL of the buffer at room temp for 12 h, followed by two successive centrifugations at 15,000 r.p.m for 5 min to separate the bound protein from the unbound. For solution depletion isotherms, IMT-CBD-mC of the range 232 µM-10 µM (at concentrations mentioned) was bound to cellulose, as mentioned above, and then unbound protein separated. A standard curve of IMT-CBD-mC fluorescence vs. the concentration determined using Bradford assay (145 µL Bradford reagent from Biorad + 5 µL protein sample) was used to measure the amount of unbound protein used. Fluorescence was measured using BioTek® hybrid fluorimeter, using endpoint measurement, with an excitation wavelength of 580 nm and emission at 610 nm. Data represents measurements made in triplicates of each point. Curve fitting was carried out using Graphpad software with non-linear regression. Bmax was calculated assuming one-site specific binding and represents amount of protein (µmol) that binds per g of cellulose.

### Crystal violet assay of biofilms

The crystal violet assay of Mtb and Msm biofilms was performed as described earlier [3]. Briefly, the crystal violet assay was performed in inkwell bottles or 24-well plates, as per requirement. After the formation of the bacterial biofilm (in inkwell bottles) the media was decanted and washed with 15 ml of 1 × PBS, and 15 ml of 1% CV was gently added to the inkwell bottles to stain the biofilm. The bottles were incubated for 20 min at 37 °C. The stain was then removed and washed twice with 15 ml of 1 × PBS. The bound crystal violet was then extracted by a 10 min incubation at 37 °C with 15 ml of 95% ethanol. After the formation of the bacterial biofilm (in 24 well plates) the media was removed and washed with 1 × PBS, and 2 ml of 1% CV was gently added to the biofilm. It was incubated for 15 min at 37 °C. The stain was removed, and the biofilm was gently washed twice with 1 × PBS. The bound crystal violet was then extracted by a 10 min incubation at 37 °C with 2 ml of 95% ethanol. The absorbance of extracted crystal violet was measured at 595 nm on a spectrophotometer. The CV assay was performed in three independent replicates for each dataset.

### Confocal laser scanning microscopy

Mature Msm biofilms were produced on chamber slides and stained with fluorescent probes such as Texas red Hydrazide (0.5 mg ml^−1^; Cat no T6256, Molecular Probes), Nile red (1 mM, Cat no N1142, Molecular Probes), Con A–Alexa Fluor 647 (200 μg ml^−1^, Cat no C21421, Molecular Probes), Calcofluor white (3 mg ml^−1^, Cat no 18909, Sigma), and IMT-CBD-mC (10 µM). Biofilms were stained with Con A for 45 min, Texas Red Hydrazide and Nile Red for 20 min, and Calcofluor white for 30 min. IMT-CBD-mC was used as a stain at 10 µM concentration in TRIS buffer (50 mM, pH 7.5) with 1 M NaCl for 30 min. After staining, samples were washed thrice with PBS. Stained biofilms were viewed using a Nikon A1R confocal microscope. For live-cell imaging of AT C58, the Cellasic ONIX2 microfluidic platform was used. Cells were cultured in TSB at 28 °C for 24 h and followed by staining with IMT-CBD-mC in TSB medium (5 µM).

### Cellulase treatment of biofilms

Msm biofilms were treated with cellulase (*T. viride*, Calbiochem) at 5 mg ml^−1^ in citrate buffer, 0.05 M, pH 4. Biofilms were treated with cellulase and citrate buffer at 0.05 M, pH 4 (kept as control) for 6 h. After treatment, biofilms were washed thrice with PBS and stained with IMT-CBD-mC for visualization of binding with cellulose. Stained biofilms were viewed using a Nikon A1R confocal microscope. The images were analyzed using NIS elements and Imaris V 9.2 software.

## Supplementary Information

**Additional file1:** **Supplementary Figure 1.** Formation of biofilm by Msm upon exposure to DTT. **Supplementary Figure 2.** 96-well plates with adherent Mtb biofilms were stained with crystal violet and quantitated for biofilm formation. The left side indicates the percentage of BSA in the O-ADC supplement added 5% to 7H9 medium, in which the bacterial cells were grown to O.D.600 of 1 and then treated in triplicates with 4, 6, and 8 mM DTT. 200 µL of the culture from the treated cells was introduced in each well and incubated at 37°C for 29 hrs. Measurement of the amount of adherent material from the thus formed biofilms was performed using CV assay for biofilm. Data represent mean ± SEM from 3 independent experiments. Statistical significance was determined using Student’s t-test, **** indicates a *p*-value of <0.0001. **Supplementary Figure 3.** 96-well plates with adherent Msm biofilms were stained with crystal violet and quantitated for biofilm formation. The left side indicates the percentage of BSA in the O-ADC supplement added to 5% to 7H9 medium, in which the bacterial cells were grown to O.D.600 of 1 and then treated in triplicates with 4, 6 and 8 mM DTT. 200 µL of the culture from the treated cells was introduced in each well and incubated at 37°C for 29 hrs. Measurement of the amount of adherent material from the thus formed biofilms was performed using CV assay for biofilm. Data represent mean ± SEM from 3 independent experiments. Statistical significance was determined using Student’s t-test. ** indicates a *p*-value of 0.01, **** indicates a *p*-value of <0.0001. **Supplementary Figure 4.** Denaturation of BSA by DTT was further validated by measuring the intrinsic fluorescence (Excitation at 280 nm) of 7H9 medium supplemented with 5% O-ADC medium (with and without tween 80) incubated at 37°C and treated with 6 mM DTT for 24h (intrinsic fluorescence was measured by sampling 200 µL sample in triplicates in a fluorescence compatible 96-well plates at indicated time points). **Supplementary Figure 5.** Msm-eGFP biofilms were stained for cellulose using IMT-CBD-mC probe (10 µM) and visualized with fluorescence microscopy (Scale bar: 20 µm).

## Data Availability

There are no large datasets in the manuscript. Data are presented in the analyzed form in the manuscript. Raw data from the current study will be available from the corresponding author on reasonable request.

## Abbreviations

EPS: Extracellular polymeric substances
Mtb: *Mycobacterium tuberculosis*
DTT: Dithiothreitol
TRS: Thiol reductive stress
BSA: Bovine serum albumin
kDa: kiloDalton
Msm: *Mycobacterium smegmatis*
O-ADC: Oleic acid, dextrose, catalase and BSA
eGFP: Enhanced green fluorescent protein
CBD: Cellulose-binding domain
PBS: Phosphate buffered saline
*C. fimi*: *Cellulomonas fimi*
AT C58: *Agrobacterium tumifaciens* C58
TSB: Tryptic Soy Broth
CV: Crystal violet
DIC: Differential interference contrast
IMT: IMTECH
