# Supplementary material for: Media component bovine serum albumin facilitates the formation of mycobacterial biofilms in response to reductive stress
Source: BMC Microbiol. 2023 Apr 20;23:111. doi: 10.1186/s12866-023-02853-6 (PMC10116703; doi:10.1186/s12866-023-02853-6)
Supplement: Supplementary file 1 — Additional file1: Supplementary Figure 1. Formation of biofilm by Msm upon exposure to DTT. Supplementary Figure 2. 96-well plates with adherent Mtb biofilms were stained with crystal violet and quantitated for biofilm formation. The left side indicates the percentage of BSA in the O-ADC supplement added 5% to 7H9 medium, in which the bacterial cells were grown to O.D.600 of 1 and then treated in triplicates with 4, 6, and 8 mM DTT. 200 µL of the culture from the treated cells was introduced in each well and incubated at 37°C for 29 hrs. Measurement of the amount of adherent material from the thus formed biofilms was performed using CV assay for biofilm. Data represent mean ± SEM from 3 independent experiments. Statistical significance was determined using Student’s t-test, **** indicates a p-value of <0.0001. Supplementary Figure 3. 96-well plates with adherent Msm biofilms were stained with crystal violet and quantitated for biofilm formation. The left side indicates the percentage of BSA in the O-ADC supplement added to 5% to 7H9 medium, in which the bacterial cells were grown to O.D.600 of 1 and then treated in triplicates with 4, 6 and 8 mM DTT. 200 µL of the culture from the treated cells was introduced in each well and incubated at 37°C for 29 hrs. Measurement of the amount of adherent material from the thus formed biofilms was performed using CV assay for biofilm. Data represent mean ± SEM from 3 independent experiments. Statistical significance was determined using Student’s t-test. ** indicates a p-value of 0.01, **** indicates a p-value of <0.0001. Supplementary Figure 4. Denaturation of BSA by DTT was further validated by measuring the intrinsic fluorescence (Excitation at 280 nm) of 7H9 medium supplemented with 5% O-ADC medium (with and without tween 80) incubated at 37°C and treated with 6 mM DTT for 24h (intrinsic fluorescence was measured by sampling 200 µL sample in triplicates in a fluorescence compatible 96-well plates at indicated time points). S [file 12866_2023_2853_MOESM1_ESM.docx]

Supplementary Material

Supplementary Figure ***1:* Formation of biofilm by Msm upon exposure to DTT: (A)** (I) Biofilms were formed in 24 well plate, the culture of O.D._600_-1.0 was exposed to 5 mM DTT (right panel). In control, no DTT was added to the culture (middle panel). To media alone, 5 mM DTT was added (left panel) (II) The graph represents the quantitation of the experiment described above using the absorbance of CV assay (**B)** (I) Biofilms were formed in 24 well plate, Msm cultures at an OD_600_ -1.0 were exposed to a 2 mM-8 mM range of DTT, and biofilm formation was recorded after 29 hrs of DTT exposure. (II) The graph represents the quantitation of the experiment described above using the absorbance of CV assay. **(C)** (I) Biofilms were formed in 24 well plate, cultures of different O.D. (0.2 – 2) were exposed to 6 mM DTT, and biofilm formation was recorded after 29 hrs of DTT exposure. (II) The graph represents the quantitation of the experiment described above using the absorbance of CV assay. **(D)** (I) Biofilms were formed in 24 well plate, Msm culture of OD_600_ - 1.0 was raised in a medium containing different concentrations of O-ADC (as indicated above). These cultures were exposed to DTT for 29 hrs. (II) The graph represents the quantitation of the experiment described above using the absorbance of CV assay. **(E)** (I) 7H9 medium was supplemented with reconstituted O-ADC having a variable concentration of BSA (concentration of BSA is denoted as % indicated above), keeping the rest of the components constant. Bacterial culture was raised in these media and exposed to 6 mM DTT. Biofilm formation was facilitated in a BSA-dependent manner. (II) The graph represents the quantitation of the experiment described above using the absorbance of CV assay. Data represent mean ± SEM from 3 independent experiments. Statistical significance was determined using Student’s *t*-test. * indicates a p-value of <0.05, *** indicates a p-value of <0.001, **** indicates a p-value of <0.0001.

**Supplementary Figure 2**: 96-well plates with adherent Mtb biofilms were stained with crystal violet and quantitated for biofilm formation. The left side indicates the percentage of BSA in the O-ADC supplement added 5% to 7H9 medium, in which the bacterial cells were grown to O.D._600_ of 1 and then treated in triplicates with 4, 6, and 8 mM DTT. 200 µL of the culture from the treated cells was introduced in each well and incubated at 37°C for 29 hrs. Measurement of the amount of adherent material from the thus formed biofilms was performed using CV assay for biofilm. Data represent mean ± SEM from 3 independent experiments. Statistical significance was determined using Student’s *t*-test, **** indicates a p-value of <0.0001.

**Supplementary Figure 3**: 96-well plates with adherent Msm biofilms were stained with crystal violet and quantitated for biofilm formation. The left side indicates the percentage of BSA in the O-ADC supplement added to 5% to 7H9 medium, in which the bacterial cells were grown to O.D._600_ of 1 and then treated in triplicates with 4, 6 and 8 mM DTT. 200 µL of the culture from the treated cells was introduced in each well and incubated at 37°C for 29 hrs. Measurement of the amount of adherent material from the thus formed biofilms was performed using CV assay for biofilm. Data represent mean ± SEM from 3 independent experiments. Statistical significance was determined using Student’s *t*-test. ** indicates a p-value of 0.01, **** indicates a p-value of <0.0001.

**Supplementary Figure 4**: Denaturation of BSA by DTT was further validated by measuring the intrinsic fluorescence (Excitation at 280 nm) of 7H9 medium supplemented with 5% O-ADC medium (with and without tween 80) incubated at 37°C and treated with 6 mM DTT for 24h (intrinsic fluorescence was measured by sampling 200 µL sample in triplicates in a fluorescence compatible 96-well plates at indicated time points). Since detergents can affect the process of aggregation of proteins, Tween-80, which is used as an anti-clumping agent at 0.01% concentration, was added as a control to test its effect on the aggregation of BSA under DTT. Medium supernatant (conditioned medium) of Msm culture at O.D. _600_ 1 was also tested and showed a similar decrease in fluorescence. Data represent mean ± SEM from 3 independent experiments. Statistical significance was determined using Student’s *t*-test. ** indicates a p-value of < 0.01, *** indicates a p-value of < 0.001, **** indicates a p-value of <0.0001.

**Supplementary Figure 5**: Msm-eGFP biofilms were stained for cellulose using IMT-CBD-mC probe (10 µM) and visualized with fluorescence microscopy (Scale bar: 20 µm).
